# A sociotechnical approach to defining clinical responsibilities for patient-generated health data

**DOI:** 10.1038/s41746-025-01680-5

**Published:** 2025-05-12

**Authors:** Ashley C. Griffin, Meagan F. Moyer, Arash Anoshiravani, Sondra Hornsey, Christopher D. Sharp

**Affiliations:** 1 Center for Biomedical Informatics Research, Stanford University School of Medicine, Stanford, CA USA; 2 Center for Innovation to Implementation, VA Palo Alto Health Care System, Menlo Park, CA USA; 3 Department of Medicine, Stanford University School of Medicine, Stanford, CA USA; 4 Stanford Health Care, Stanford, CA USA; 5 Deparment of Pediatrics, Stanford University School of Medicine, Stanford, CA USA; 6 Stanford Medicine Children’s Health, Stanford, CA USA; 7 Vanderbilt University Medical Center, Nashville, TN USA

**Keywords:** Health care, Health policy

## Abstract

The proliferation of health devices and apps has led to an abundance of patient-generated health data (PGHD), which has raised concerns about integration within clinical settings. We describe one health system’s sequential focus group approach for developing guiding principles to inform clinical responsibilities of PGHD. These principles center around (1) setting expectations; (2) preparing staffing and workflows; (3) delivering high-quality experiences; and (4) considerations for health information management of PGHD.

## Introduction

Several major clinical informatics advancements are aligning to incorporate patients’ personal health data and experience into care settings with immense potential to improve health outcomes. Estimates indicate 90% of U.S. adults own a smartphone^1^, and over 50% use health apps to monitor their health^2^. The rise of consumer health technologies presents opportunities for patients and caregivers to communicate their health-related data with their care team. Patient-generated health data (PGHD), or health data created and recorded by patients outside of the clinical setting^3^, has demonstrated early value in improving health outcomes and supporting personalized medicine^4,5^. For example, use of PGHD has been associated with improved physiologic measures and symptoms in both adult and pediatric patients for a variety of conditions such as diabetes, asthma, and cancer^5–7^. This early evidence suggests that some types of PGHD may be used as an adjunct to traditional forms of care.

Much progress has also been made in the regulatory and interoperability landscape around collection and use of PGHD. In the U.S., several Current Procedural Terminology (CPT) codes were created in 2018 for remote monitoring of physiologic measures (e.g., blood pressure, pulse oximetry, weight)^8,9^. The Assistant Secretary for Technology Policy and Office of the National Coordinator’s Health Information Technology Certification Program supports PGHD capture and use by requiring certified electronic health records (EHRs) to identify, store, and access information electronically shared by a patient^10^. Health data exchange has been accelerated by Health Level Seven’s Fast Healthcare Interoperability Resources (FHIR) standard, which supports interoperability between health information technology systems, such as mobile apps and EHRs^11^. This makes it possible to integrate third-party app data into EHRs and for patients to access their health records through apps like Apple Health and Android CommonHealth which use FHIR. Notably, as of 2022, a FHIR Application Programming Interface is now a requirement for EHR certification as part of the 21^st^ Century Cures Act^12^, which allows developers to build innovative tools that can help patients track, use, and share their health information. Beyond the U.S., the 2024 “State of FHIR” global survey results indicated that 23 out of 29 countries have regulations for the use of standards in electronic health data exchange, with over half reporting regulations that specifically mention FHIR^13^. Numerous global initiatives also focus on improving use of personal health data and PGHD, such as the European Union’s InteropEHRate^14^, Patients Know Best Initiative in the UK^15^, and India’s National Digital Health Blueprint^16^. Importantly, the Global Health Digital Partnership, a collaborative effort between 40 countries and the World Health Organization, supports implementation of digital health services and interoperability^17^.

As the use of PGHD and remote monitoring programs continues to grow in clinical care models, questions concerning clinical accountability, liability, and workflow integration of PGHD have arisen for many healthcare organizations^18–20^. Health system leaders have highlighted the limited evidence around the clinical significance and thresholds for intervening based on PGHD^21^. Clinicians have also expressed concerns about data quality and accuracy of manual PGHD entry, such as fingerstick blood glucose measurements^18,22^. One study found that presenting PGHD with clinic laboratory data may cause confusion for clinicians due to differences in reliability and completeness^22^. Additional issues have emerged around the time burden to review and discuss PGHD with patients^23^. Both patients and health systems have felt it was important for care teams to explain how PGHD was being analyzed for clinical decision-making^21^. With the wide range of PGHD data streams, questions have arisen on topics related to privacy, security, and data integration with the legal medical record^24^. Particularly for passive data collection, where biometric data can be collected every second, there are concerns around the frequency of data collection, transfer, storage, and how these high-volume data can be meaningfully represented and used for clinical care within the EHR^25^. As a result, care teams have reported the need for protocols that guide health care staff decisions and establish guidelines for patients around how their data is used, when data is reviewed, and when they might be contacted^26^.

Given the rising questions and need for guidance on the use of PGHD to inform patient care and delivery, we describe one health system’s approach to creating guiding principles that inform clinical responsibilities and expectations for PGHD programs. This commentary outlines our sociotechnical approach, common themes from workgroup sessions, and recommendations to guide clinical, technology, and administrative teams on PGHD policies and standards.

## Approach

### Setting and sampling

Within an academic medical center (Stanford Medicine), a core group was founded to examine the clinical and legal responsibilities of care teams in the receipt, analysis, and usage of PGHD. The group consisted of a Chief Medical Information Officer, Health Information Management Systems (HIMS) Medical Director, Associate Privacy Officer, Health Care Digital Health Operations Consultant, and Medical Informaticist. The core group planned the scope and content of the work, hosted workgroup sessions, and drafted, edited, and disseminated the final PGHD guiding principles. Convenience sampling was used to identify workgroup session participants with expertise in PGHD, HIMS, privacy, and compliance. For clinical participants, we identified clinicians with varying medical specialties and patient populations (i.e., pediatric and adult populations) who had experience using PGHD.

### Patient-generated health data scenarios

**Fig. 1:**
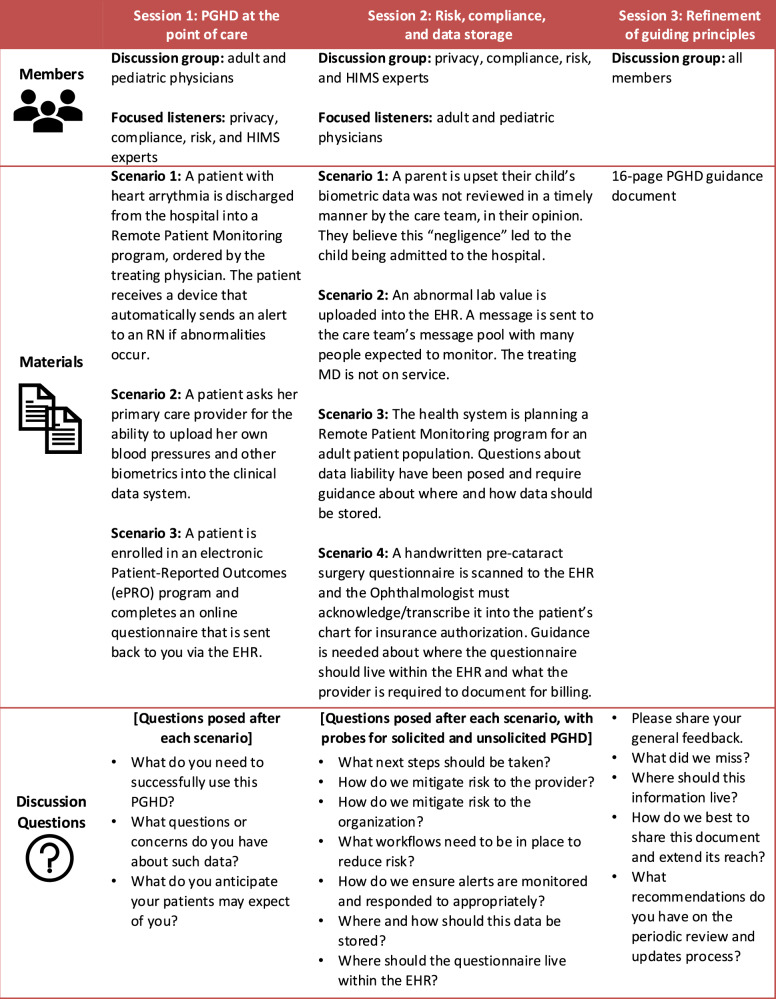
Patient-generated health data workgroup session members, materials, and discussion questions. Workgroup session topics focused on (1) PGHD at the point of care, (2) risk, compliance, and data storage, and (3) refinement of the guiding principles. Members in each session were represented as part of the core discussion group or as focused listeners. Scenarios and open-ended discussion questions were intended to elicit perspectives and interactive discussions on members’ experiences with PGHD.

The core group developed scenarios that were guided by an 8-dimension sociotechnical model^27^ to understand the relationship between health information technology, clinicians, and workflows (Fig. 1). For example, scenarios and open-ended discussion questions were created to elucidate the communication structures and staffing needs for PGHD implementation, which pertain to the “workflow and communication” and “people” dimensions in the sociotechnical model. Other scenarios were related to mitigating organizational risk and periodic review of the guiding principles, corresponding to the “organizational policies and procedures” and “external rules and regulations” dimensions. Scenarios were written to consider a variety of clinical conditions, specialties, and PGHD types (i.e., solicited data, unsolicited data, questionnaire data). We defined solicited data as data provided by the patient as requested from the care team either as part of a remote monitoring program or ad hoc request from the clinician, whereas unsolicited data was defined as data provided by the patient to the care team without solicitation from a clinician.

### Workgroup sessions

Three sequential workgroup sessions (i.e., focus groups) were conducted to foster interactive discussions on participants’ experiences with PGHD. The goal of the sessions was to generate rich insights and perspectives on incorporating PGHD into clinical care. These sessions were highly participatory and discussion-focused and took place between March and June 2022. Sessions were iterative in nature such that each session informed the next.

During the first session, three clinical scenarios were presented to the physician workgroup members to elicit feedback. Throughout the first session, physician members comprised the discussion group, and members from privacy, compliance, risk, and health information management attended as focused listeners. Findings from the first session were then presented during the second workgroup session to receive input from privacy, compliance, risk, and health information management members, with the physicians as focused listeners. Different risk management scenarios based on the themes from the first session were presented with probes for how the group would respond to solicited data versus unsolicited data. Feedback from both the first and second sessions was used to draft the PGHD guiding principles. Lastly, the third workgroup session involved all members providing input on the draft of the guidance document. Open-ended questions were posed to elicit feedback on the content, organizational management, and recommendations on dissemination, periodic review, and update process.

### Analysis

Workgroup sessions were video and audio recorded over Zoom, and detailed notes were taken during each session. After each session, a summary of the notes was distributed to participants who were asked to review and clarify the notes as necessary. Between each session, the core group conducted rapid qualitative analysis^28,29^, as our focus was on swiftly addressing real-world challenges of integrating PGHD into clinical care. The core group reviewed the recordings paired with the notes and summarized data based on discussion question topics. Summaries were then synthesized and grouped into themes through additional collaborative discussions, revisiting the recordings when needed. To establish rigor and trustworthiness of the analysis, the themes were presented at the beginning of the workgroup sessions, and participants were asked for input on the validity of the themes. Participants expressed that the themes accurately represented their views. Informed by the sociotechnical model, themes across each session were harmonized to create the PGHD guiding principles. For example, related “workflow and communication” themes arose in the first and second sessions regarding workflows for clinical care and workflows for risk mitigation. Thus, these themes were grouped together into a single guiding principle.

## Key findings

### Participant characteristics

A total of 16 participants were engaged in the workgroup sessions, including 10 clinicians and 6 non-clinician experts from HIMS, compliance, digital health care integration, and risk management departments at the academic medical center. Clinical specialties included gastroenterology, hospital medicine, internal medicine, general surgery, clinical informatics, cardiology (adult and pediatric), pediatric endocrinology, pediatric nephrology, and pediatric adolescent medicine. Participants had varying experience with PGHD, ranging from basic familiarity to routinely using it in remote monitoring programs.

### Themes for defining clinical responsibilities with patient-generated health data

The first workgroup session yielded themes related to expectations, staffing and workflow needs, and ease of use of PGHD (Table 1). Clinicians indicated that setting expectations between care teams and patients was essential to communicate how PGHD was being used and monitored. For remote monitoring programs, clinicians discussed defining appropriate responsibilities and communicating those to patients, such as describing how data is currently not intended for real-time monitoring. However, if patients felt concerned about abnormal values, it was recommended that they call their care team. Similarly, one clinician suggested setting expectations for weekly reviews of global trends in blood glucose data. This was due, in part, to potential data quality issues of individual high and low data points. Discussion related to staffing and workflow needs centered around the challenges of having an infrastructure and workflow to respond in real-time. Clinicians emphasized the need for further training and support to respond to and manage the data, particularly for high-risk conditions and sensitive questionnaires. For data ease of use, clinicians desired triggers in the EHR to alert clinical staff for action, which might involve calling the patient. It was also desirable to view data as trends as opposed to discrete data points and to make these trends available patients to foster engagement.

**Table 1:** Themes related to patient-generated health data across workgroup sessions

Themes	Description	Representative quotes
**Session 1: PGHD at the Point of Care**		
Expectation setting between patients, care teams, and organization	• Discuss with patients how data is being used and monitored • Set expectations of patients for the response time to messages or data • Outline responsibilities of patients/caregivers and care teams • Consider clinical relevance and downstream impact of accepting unsolicited PGHD • Emphasize response to high-risk conditions and sensitive questionnaires (e.g., PHQ-9)	• *“We need to set expectations around what can be accomplished in the outpatient setting for monitoring the data, turnaround time, and ongoing follow-up.”* • *“Patients and doctors often have different ideas of what is urgent. This is why it is so important to outline responsibilities.”*
Staffing and workflow needs to successfully manage data	• Organizations should provide PGHD training for clinic staff and care teams • Personnel and workflow support are needed to respond to and monitor data, typically in the scenario of solicited PGHD (i.e., remote monitoring program) • Workflows should consider treating providers not being present • Consider reconciliation of data (biometrics, questionnaires, other data sources flowing in)	• *“Programs need to have support structure and adequate staffing to be successful and clinically relevant…if workflows are too complicated the program will devolve.”* • *“One of the biggest challenges is having the infrastructure to respond in real-time and having reliable response mechanisms, particularly for abnormal values.”*
Data ease of use for patients and care teams	• Data should be easy to find and use within the EHR • Data should be viewable as trends, as opposed to discrete data points • Set triggers that alert care team for action • Provide summaries of data for patients	• *“It’s a lot of extra work to review more and more data. More clicks are cumbersome and add up…We should be able to navigate to the data and review it easily.”* • *“I typically look at weekly trends instead of discrete numbers [for biometric data], unless the patient is not doing well. It would also be nice to have trends in the questionnaire data.”*
**Session 2: Risk, Compliance, and Data Storage**		
Care team and organizational risk management	• Clinical workflows must be in place to respond to PGHD that is high risk and after hours • Clinical staff should be trained on appropriate escalation pathways because normal, abnormal, and critical PGHD values differ from standard lab ordering protocols and triggers • PGHD has many different sources, and it is not possible to review or be responsible for all types of data • Document patient understanding and informed consent in the clinical progress note	• *“During the PGHD initiation process, patients and caregivers should fully understand and acknowledge the expectations of how the data is reviewed, responded to, and used, ideally through verbal and written documentation.”* • *“A workflow for sending alerts and addressing abnormal values from PGHD during off hours needs to be in place when accepting PGHD as a part of routine clinical care. All interactions with the patient should be notated in the patient’s medical record.”*
Data storage	• Retain PGHD for as long as medical records (i.e., 7 years) • PGHD should be easy to find and can be used across different specialties • PGHD storage inside vs. outside of patient’s medical record depends on how data is used to inform care • Consider how data flows in and out of the EHR as it pertains to interoperability	• *“PGHD should be housed in a standardized location within the EHR for continuity across our organization.”* • *“Storing [PGHD] in the primary medical record or secondary data repositories depends on how it is used for decision-making as well as technical capability and workflow needs.”*
**Session 3: Refinement of PGHD Guiding Principles**		
Organizational ownership	• HIMS committee accepted ownership and responsibility of the guidance document	*“There must be clear ownership to address edits and feedback…HIMS may be reasonable given their scope of overseeing health information and technology management issues and partnership with Compliance, Privacy, IT, and Risk Management departments.”*
Frequency of review	• Review annually at a minimum • Policy, regulatory, or institutional changes may necessitate more frequent review	“*Due to the ever-changing nature of technology, medicine, and regulation, the guidance should be updated on a timely basis to reflect the needs of our patients, providers, and organization.”*
Dissemination	• Leverage multiple channels across organization (i.e., internal website, organization-wide email, newsletter, departmental and committee meetings, Grand Rounds) • Develop one-page document to summarize guidance and how it should be used	“*Overcommunicating will extend the document’s reach…Distribution could be through an intranet announcement, organization-wide email, and departmental meetings.”*

The second workgroup session generated themes for risk management and storage of PGHD. Privacy and compliance experts underscored how clinical workflows must be in place to respond to PGHD that is high risk, such as an abnormal blood glucose reading in a patient enrolled in a diabetes remote monitoring program. Similarly, workgroup members recommended creating guidance and then training staff on appropriate escalation pathways. They cautioned against relying on PGHD monitoring systems to escalate the medical need. Members also felt documenting patient understanding and informed consent in the clinical progress note was important for risk management. There were differing views on where the data should be stored, particularly for solicited vs. unsolicited data, but most agreed that the portions of PGHD used for clinical decision-making should be stored within the patient’s medical record. Considerations for data storage were also related to retaining data for as long as medical record data (i.e., 7 years) and for how data flows in and out of the EHR as it relates to interoperability.

**Fig. 2:**
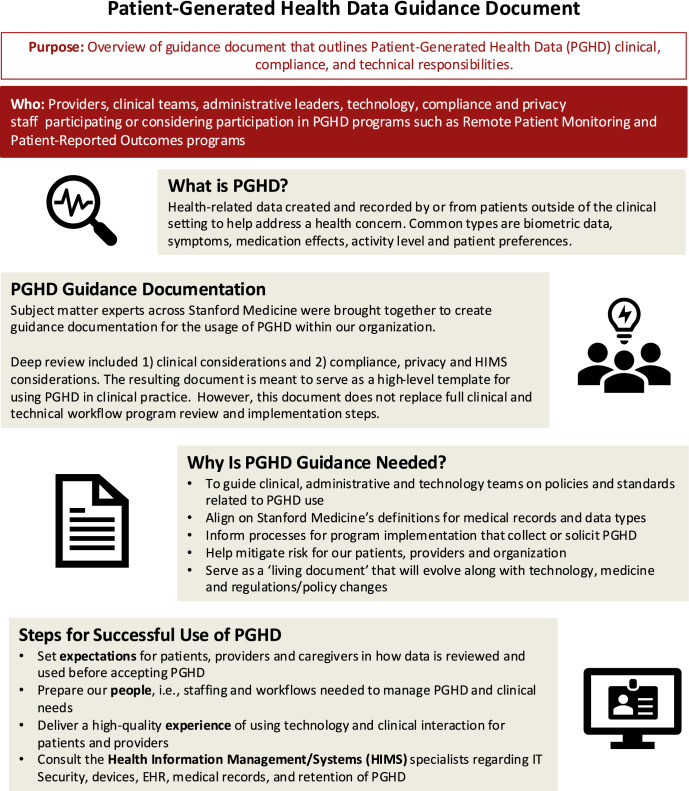
Patient-generated health data guidance document summary. The document was used to aid in dissemination of the final PGHD guidance document within the academic medical center. It describes development and rationale for the guidance, as well as highlights steps for successful use of PGHD. These steps closely align with our overarching guiding principles to guide clinical, technology, and administrative teams on PGHD policies and standards.

Upon review of the PGHD guidance document among all members, the third workgroup session resulted in themes related to organizational ownership, frequency of review and updates, and dissemination. The majority felt that the document should be owned and maintained by the health care organization’s HIMS committee. The document spanned many domains and departments, including Compliance, Risk Management, Privacy, and IT Security, with the HIMS committee already partnering with these departments to oversee all health information and technology management issues across the organization. Members also recommended that the document have an annual review process, unless there were policy, regulatory, or institutional changes that would require a more frequent review. Members encouraged disseminating the document widely through different channels, such as internal websites, organization-wide email, and departmental meetings. A one-page summary of the document was also developed to provide an overview of the guidance document, summarize steps for successful use of PGHD, and provide access the full document (Fig. 2).

## PGHD guiding principles

The key findings and themes from each workgroup session culminated in the following guiding principles to support clinical, administrative, and technology teams on PGHD-related policies and standards. The principles are intended to align health system teams on common definitions pertaining to PGHD and inform processes for the development and implementation of programs that collect and use PGHD. The following principles also seek to mitigate risk for patients, care teams, and the organization when integrating PGHD in clinical settings.

1. Set expectations for patients, caregivers, and care teams in how data is reviewed and used before accepting PGHD. For both solicited and unsolicited PGHD, it is important that the patient and/or caregiver understand how data will be monitored, viewed, responded to, and used by the care team. Patient and care team expectations should include aspects of consent (pediatric and adult patients), privacy, data amendments, and secondary uses of PGHD.
2. Plan and prepare staffing and workflows needed to manage PGHD and clinical needs. Due to differences in patient populations and care team staffing, clinics need to establish workflows that consider all aspects of patients’ and care teams’ needs in generating and using PGHD. Care teams should consider setting outlier data points that trigger action, which should be responded to based on clinical judgment and established protocols.
3. Deliver a high-quality experience of using technology and clinical interaction for patients and care teams alike. Programs that generate PGHD benefit from data being visualized to monitor values, which provides an opportunity to educate and engage patients in their care and treatment plan. It is also valuable to partner with patient advocates and advisors when creating PGHD clinical workflows.
4. Consult with health information management specialists regarding security, devices, EHR, medical records, and data retention of PGHD when planning and operating programs. Devices and apps that integrate with the EHR or patient portal should be reviewed for privacy and security before integration. Data provenance and data storage in the primary medical record or secondary data repositories should also be explicitly outlined.

Based on the above guiding principles, a PGHD clinical and operational document was created and organized in a question-answer format to provide guidance within the academic medical center. A summary of the document was used as a communication tool within the health system to aid in dissemination (Fig. 2). The full guiding document also provides definitions, information for creating a PGHD program, case studies, and related policies and procedures, which draws upon existing PGHD practice guides^23,24,30,31^. It is intended to serve as a “living document” that will evolve along with technology, medicine, and policy changes.

## Limitations

While development of the PGHD guiding principles involved input across multiple specialties and two organizations (Stanford Health Care and Stanford Medicine Children’s Health), they are specific to one health systems’ operational needs and clinical programs and may not generalize to other health systems. In addition, our approach has not been trialed at other health systems or sites, which may vary based on stakeholders, policies, and patients. The workgroup sessions involved subject matter experts, and our future work seeks to gather patient input and development of accompanying patient-facing guiding principles and educational material to support patients’ use of PGHD.

## Conclusion

As the demand for clinical integration of PGHD grows, protocols are essential to guide health care staff in the collection and use of newer data sources. These guiding principles outline the clinical and legal responsibilities of care teams in the receipt, analysis, and usage of PGHD. Our approach and guiding principles can be used by other healthcare organizations in the development and implementation of PGHD programs that meet the unique needs of their patient populations.

## Data Availability

No datasets were generated or analysed during the current study.
